# Ultrasound-based deep learning radiomics for the differential diagnosis of benign and malignant subpleural pulmonary lesions

**DOI:** 10.3389/fonc.2026.1786674

**Published:** 2026-03-16

**Authors:** Liyan Wei, Jingtong Zeng, Yi Feng, Xinhong Liao, Hong Yang

**Affiliations:** Department of Ultrasound, First Affiliated Hospital of Guangxi Medical University, Nanning, China

**Keywords:** deep learning, interpretability, radiomics, subpleural pulmonary lesions, ultrasound

## Abstract

**Objective:**

This study aims to develop an ultrasound-driven clinical deep learning radiomics (CDLR) model for the differential diagnosis of benign and malignant subpleural pulmonary lesions (SPLs), with the goal of guiding personalized treatment and minimizing unnecessary interventions.

**Methods:**

A retrospective analysis was conducted on 609 SPL patients from July 2020 to February 2024 at Guangxi Medical University. The dataset was divided into training (487 cases) and validation (122 cases) cohorts. Prior to ultrasound-guided lung mass biopsy, 1561 radiomics (Rad) features were extracted from every ultrasound image, alongside 128 deep transfer learning (DTL) features after dimensionality reduction and compression based on ResNet-50. Feature selection was performed, followed by the development of a deep learning radiomics (DLR) model using a Support Vector Machine (SVM), which was then used to derive the model’s feature. Clinical data were analyzed through univariate and multivariate logistic regression, generating the clinical features. The DLR and clinical features were integrated using SVM to create the CDLR model for differentiating benign and malignant SPLs. Model performance was evaluated using the area under the receiver operating characteristic curve (AUC), and its clinical utility was assessed *via* decision curve analysis (DCA). The Shapley Additive Explanation (SHAP) method and Gradient weighted Class Activation Mapping (Grad-CAM) visualization were employed to enhance model interpretability.

**Results:**

The CDLR model demonstrated high accuracy in distinguishing benign and malignant SPLs. The AUC values for the training and validation set were 0.987 and 0.924, respectively. Notably, the CDLR model outperformed clinical, standalone Rad, DTL, and DLR models in the validation cohort. The model also achieved the highest sensitivity (0.871), specificity (0.897), and accuracy (0.877). Grad-CAM visualization highlighted key regions of interest within ultrasound images, and SHAP analysis identified the contributions of clinical, deep learning, and radiomics features.

**Conclusion:**

The ultrasound-based CDLR model provides a robust tool for differentiating benign and malignant SPLs, offering superior diagnostic performance compared to existing ultrasound diagnostic criteria. This model is valuable for early lung cancer screening and can reduce unnecessary biopsies or surgeries for pulmonary masses.

## Introduction

Lung cancer is the leading cause of cancer-related deaths globally, with its high incidence and poor prognosis posing a continuous threat to public health. According to the 2022 Global Cancer Statistics Report, lung cancer is the most prevalent cancer worldwide (1). In China, a country with a significant lung cancer burden, both the incidence and mortality rates exceed the global averages (2). Due to insufficient early screening, approximately 75% of patients are diagnosed at advanced stages (3). The 2024 NCCN Lung Cancer Screening Guidelines emphasize that early and accurate diagnosis is crucial for improving prognosis (4). However, current screening methods continue to face several challenges.

Traditional diagnostic approaches for lung cancer, such as biopsy, Low-Dose Computed Tomography (LDCT), and PET-CT, present limitations, including radiation exposure, complex procedures, high costs, and biopsy risks (5–7). Subpleural pulmonary lesions (SPLs), located adjacent to the visceral pleura and clearly visible *via* ultrasound, benefit from ultrasound’s advantages: no radiation, real-time dynamic imaging, and high repeatability. This has made ultrasound a promising tool for SPL assessment (8, 9). However, traditional ultrasound diagnoses heavily rely on the physician’s experience, which introduces subjectivity. Thus, the development of quantitative ultrasound image analysis methods is crucial to minimize such biases.

The integration of radiomics and deep learning enhances medical image analysis by combining manually extracted features with deep transfer learning (DTL) features, overcoming the limitations of single-modality approaches and improving diagnostic accuracy. For example, a CT-based model utilizing deep learning radiomics features has achieved high sensitivity and specificity in diagnosing pulmonary nodules (10, 11),while The ultrasound-based radiomics machine learning model can effectively predict the cervical lymph node metastasis of lung cancer (12). However, studies exploring the use of DTL in conjunction with ultrasound radiomics for differentiating benign and malignant SPLs remain limited.

Therefore, this study aims to leverage ultrasound technology, deep learning, and radiomics to develop a machine learning (ML) model for the non-invasive differential diagnosis of benign and malignant SPLs. Furthermore, this study utilized the Gradient weighted Class Activation Mapping (Grad-CAM) technique to visually mark the areas of the model that receive the most attention (13), and applied Shapley Additive Explanation (SHAP) to explain the contribution values of the features in complex ML models (14), thereby assisting doctors in making the most appropriate clinical decisions.

## Materials and methods

### Study population

**Figure 1:**
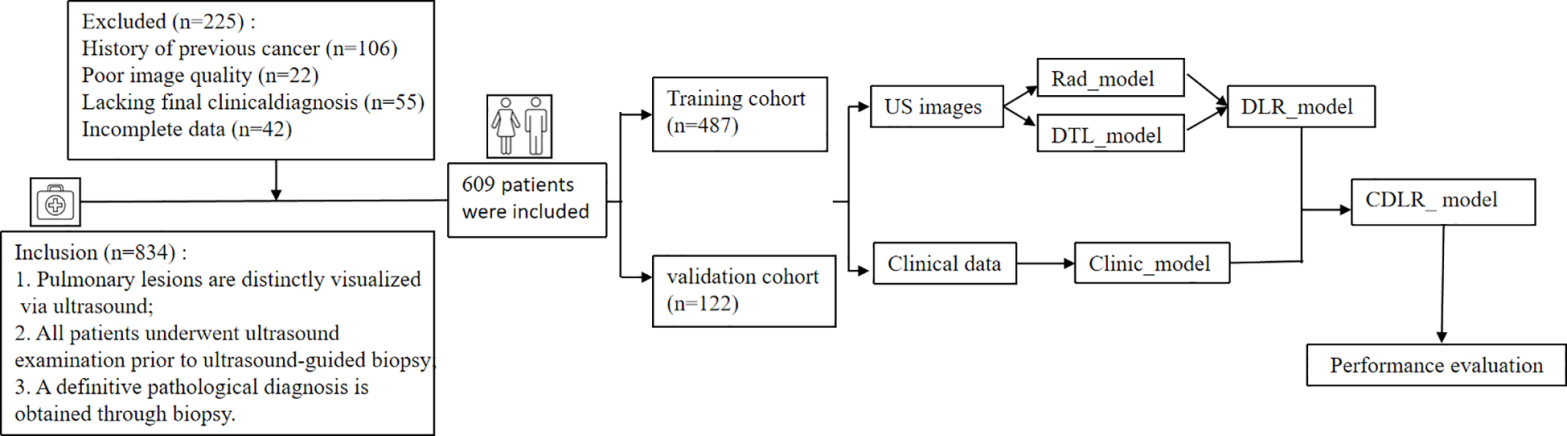
Patient selection process for this study depicted in a flowchart.

A retrospective study was conducted on patients who visited the First Affiliated Hospital of Guangxi Medical University and were diagnosed with SPLs *via* CT examination from July 2020 to February 2024. This study was accordance with the ethical standards formulated in the Helsinki Declaration, all data were anonymized and did not involve the leakage of patient privacy or additional intervention. The study was officially reviewed and approved by the Medical Ethics Committee of the First Affiliated Hospital of Guangxi Medical University, which exempted the need for informed consent from the subjects.(approval number: 2025-E0979). After applying the inclusion and exclusion criteria, 609 patients with SPLs were included. Of these, 422 were diagnosed with malignant conditions based on pathological results, while 187 were confirmed as benign by puncture pathology. Meanwhile, the clinicians diagnosed them as benign lesions based on the results of imaging analysis, serological tests, and sputum culture. The distribution of lesion pathology types is provided in **Supplementary Table E1**. Before all the feature extraction, feature selection, model parameter adjustment, or any step involving training the model using data, we first used random sampling to independently divide 609 patients into a training set (n = 487) and a validation set (with n = 122) in an 8:2 ratio. The study flowchart is shown in **Figure 1**.

### Clinical data

Clinical data, including demographics (age, gender, smoking history) and serum markers (CA125, CYFRA21-1), were collected from the medical record system, within one month prior to biopsy.

### Ultrasonic image acquisition

In this study, all the research subjects underwent routine two-dimensional ultrasound examinations using ultrasound instruments equipped with 2–8 MHz convex array probes. The equipment mainly included GE LOGIQ E9, Mindray Resona R9, My Lab Class C, and Hitachi HI VISION Ascendus. The scanning depth was set to 5–17 cm based on the size of the lesion. The total gain was adjusted to 20-80, and the dynamic range was controlled within 20–90 dB. Images with obvious blurring or artifacts were immediately re-acquired. All the images finally included in the analysis met the quality control standards. The process of acquiring ultrasound images of SPLs is detailed in **Supplementary Material E2**. Images were retrieved from the unified image storage system of the First Affiliated Hospital of Guangxi Medical University: Picture Archiving and Communication System (PACS) and exported in JPG format. While JPG’s 8-bit depth and lossy compression cause information loss compared to original DICOM (12–16 bits), this limitation has minimal impact on our 2D ultrasound analysis. Lesion morphology and edge features remain clearly distinguishable in JPG format. Pixel spacing, set based on ultrasound equipment specifications, has limited effect on texture feature extraction. In addition, All acquired JPG images were uniformly converted to NIfTI format (.nii.gz) using SimpleITK library. The conversion process preserved the original image resolution and spatial information, guaranteeing the accuracy of subsequent data analysis and ROI delineation. To ensure comparability and image quality, all patients were examined by ultrasound doctors with more than five years of experience. For consistency, the maximum cross-sectional view of each lesion was selected for analysis. In cases with multiple lesions, the lesion targeted for biopsy was chosen as the focus of the study.

### Image segmentation and feature extraction

All images were imported into Insight Segmentation and Registration Toolkit SNAP (ITK-SNAP) software (version 3.8; http://www.itksnap.org), where the regions of interest (ROIs) for each lesion were manually delineated by two sonographers with 5 years of experience, tracing the lesion boundaries. To ensure reliability, the reproducibility of the extracted features was evaluated using intraclass correlation coefficients (ICCs). For this, 30 images were randomly selected, which were re-analyzed by an ultrasound specialist with 8 years of experience to assess inter-observer consistency. The ICC value for all image features of the two physicians was 0.977(95% CI: 0.953 to 0.989), which were all greater than the clinical research acceptable threshold of 0.75. all specialists were blinded to the clinical information and pathological results of the patients.

Based on the manually segmented region of interest (ROI), the radiomics and DTL features of the images were extracted using the Pyradiomics software package in Python (version 3.7.12). These features mainly include shape features, first-order features, and texture features, totaling 1561 radiomics features. Based on ResNet-50, the deep transfer learning feature extraction process consists of four steps: data preprocessing, model loading and adjustment, feature extraction, PCA feature compression and dimensionality reduction. Finally, 128 feature vectors corresponding to the maximum eigenvalue were retained to improve the generalization ability of the model and reduce the risk of overfitting. For the specific PyRadiomics configuration, the detailed distribution of radiomics features, and the comprehensive information on deep learning feature extraction, please refer to **Supplementary Material E3**.

### Feature selection

This study employed a multi-stage screening mechanism for radiomics and deep learning features: Firstly, features with an ICC > 0.75 were excluded based on their low repetition rate; Secondly, all remaining features were standardized using Z-score, and variables with significant differences between benign and malignant lung lesions (p < 0.05) were selected through t-test or Mann-Whitney U test; Thirdly, redundant features with strong correlations were eliminated using Pearson correlation coefficient (threshold > 0.9); Finally, features with non-zero coefficients were obtained through LASSO regression (5-fold cross-validation). It is worth noting that to strictly prevent data leakage in the machine learning process, all operations involving parameter fitting are restricted to the training set. The validation set is only used for the final performance evaluation of the model after all training and tuning are completed. Throughout the process, it has not been used for model selection, hyperparameter adjustment, or feature reselection, ensuring its unbiased nature as an independent evaluation set.

Logistic regression was applied to clinical variables, with variables showing *p* < 0.05 selected for multivariate analysis to identify key predictors for the clinical model. This process generated odds ratios (OR) and 95% confidence intervals, leading to the final clinical feature.

### Establishment of CDLR

**Figure 2:**
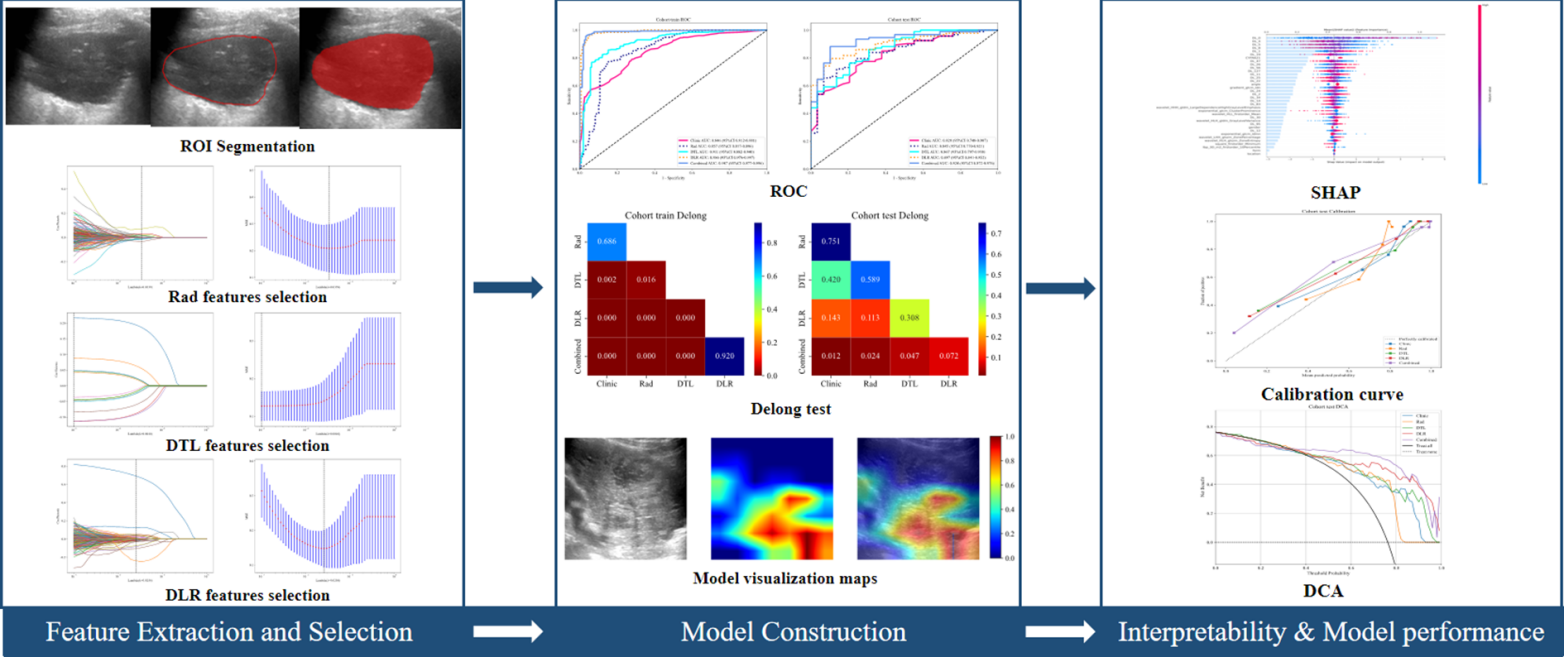
Rad, DTL, DLR and CDLR workflow.

To integrate clinical and DLR features into a decision-support model, SVM was used to develop the combined clinical deep learning radiomics (CDLR) model. The efficacy of the model was evaluated using ROC curves in both training and validation cohorts, yielding area under the curve (AUC), accuracy (ACC), sensitivity (SEN), specificity (SPE), positive predictive Value (PPV), and Negative Predictive Value (NPV). Delong tests were conducted to compare AUC differences (*p* < 0.05), confirming the robustness of the CDLR model. The contribution and interpretability of CDLR model were assessed using the SHAP method. The workflow is outlined in **Figure 2**.

### Statistical analysis

All data analyses were performed using the OnekeyAI platform version 4.9.1, with Python 3.7.12 for comprehensive statistical analysis. Continuous variables were analyzed using t-tests or Mann-Whitney U tests, depending on their distribution, while categorical variables were examined with chi-square tests. A two-sided *p* < 0.05 was considered statistically significant.

## Results

### Baseline characteristics

Patients**’** clinical data and characteristics are summarized in **Supplementary Material Table E4**. The study included 609 patients with SPLs, comprising 372 males (61.1%) and 237 females (38.9%). Of these, 187 cases were benign, and 422 cases were malignant.

### Construction and validation of Rad, DTL, and DLR

**Figure 3:**
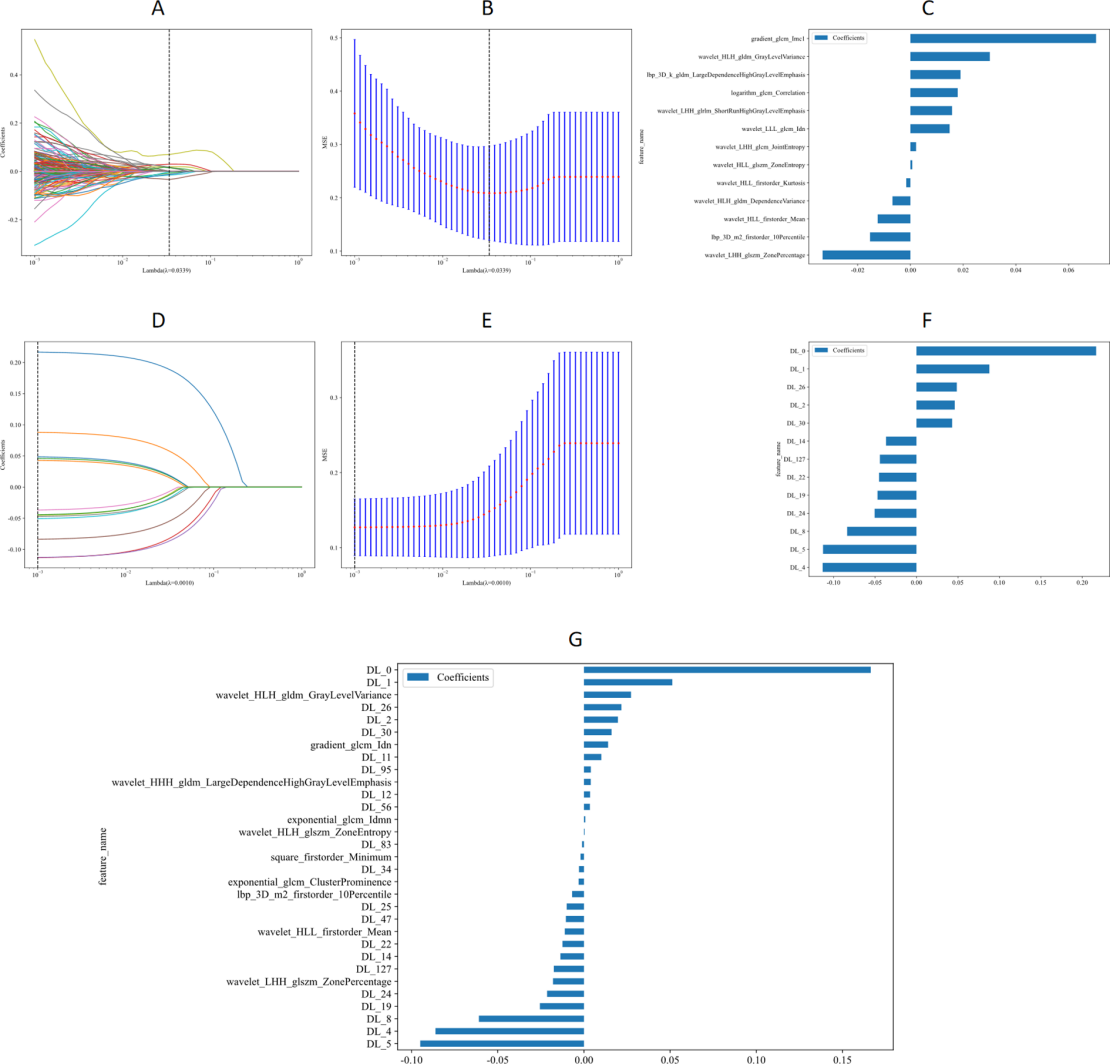
LASSO and MSE of fivefold cross-validation. **(A, D)** the coefficients of the Rad and DTL features obtained through LASSO. **(B, E)** MSE from the five-fold cross-validation process. **(C, F)** the coefficients of the filtered Rad and DTL features, while panel **(G)** illustrates the coefficients of the final DLR features after filtering. LASSO, Least Absolute Shrinkage and Selection Operator; MSE, Mean Squared Error; Rad, Radiomics; DTL, Deep Transfer Learning; SPL, Subpleural Lung Lesions; DLR, Deep Learning Radiomics.

To differentiate between benign and malignant SPL lesions, a DLR model was constructed using 13 Rad features and 13 DTL features, resulting in the development of 31 DLR features (**Figure 3**).

### Development and validation of clinical model and CDLR

In the training cohort, five independent predictors were identified: gender, lesion anatomical location, angle, presence of bronchial signs within the lesion, and CYFRA21-1 (**Table 1**), which influenced the differentiation between benign and malignant SPLs. Using these factors, a clinical model was constructed, forming the clinical feature.

**Table 1:** Univariate and multivariate logistic regression analysis.

Characteristic	Univariate logistic regression analysis		Multivariate logistic regression	
	*OR (95%CI)*	*p-value*	*OR (95%CI)*	*p-value*
Age	1. 015 (1. 012, 1.017)	<0.001	1.000 (0.989, 1.01)	0. 945
Gender*	1. 156 (1.125, 1. 188)	<0.001	1.026 (0. 924, 1. 138)	0.691
Smoking history	1. 224 (1.179, 1.269)	<0.001	1. 130 (0. 983, 1. 297)	0. 148
location*	2. 300 (1.868, 2. 832)	<0.001	0. 338 (0.186, 0. 614)	0.003
Transverse diameter	1.193 (1.134, 1. 254)	<0.001	0. 663 (0.580, 0. 759)	<0.001
Vertical diameter	4. 451 (3. 449, 5. 743)	<0.001	2. 165 (1. 221, 3. 838)	0. 026
form	4. 030 (3. 216, 5.053)	<0.001	2. 386 (1. 335, 4. 263)	0. 014
Angle*	1. 325 (1. 034, 1. 697)	0.063		
Bronchial sign*	2. 534 (2.061, 3. 117)	<0.001	0.822 (0.529, 1. 274)	0. 461
blood	2. 687 (2.113, 3. 418)	<0.001	1.437 (0. 798, 2. 588)	0. 311
CA125	3.800 (2. 883, 5. 008)	<0.001	1.663 (1. 082, 2. 555)	0. 052
CYFRA21-1*	7. 516 (5. 490, 10. 299)	<0.001	7.552 (4.855, 11. 752)	<0.001

*For log; *OR*, odds ratios; *CA125*, Cancer Antigen 125; *CYFRA21-1*, Cytokeratin 19 Fragment 21-1.

**Figure 4:**
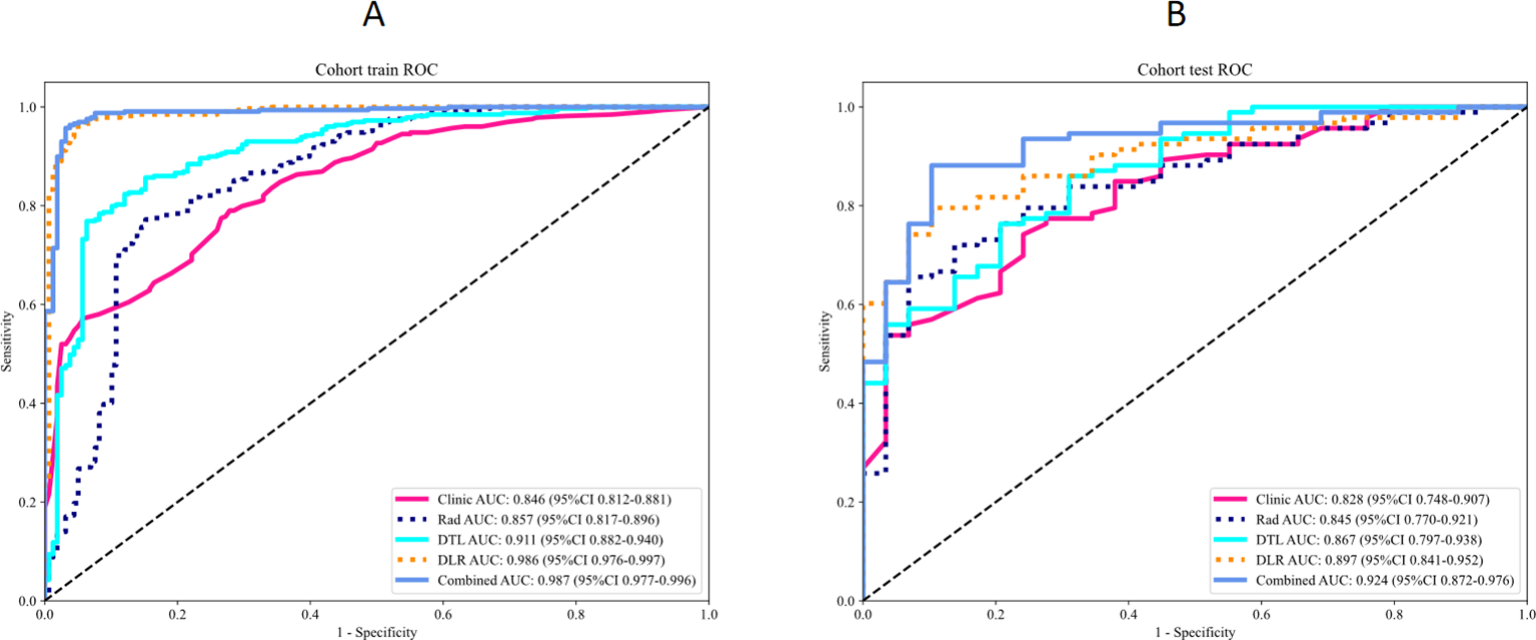
ROC curves compare the performance of the clinical, Rad, DTL, DLR and CDLR models in the training set and validation set. ROC, Receiver Operating Characteristic; Rad, radiomics; DTL, Deep Transfer Learning; DLR, Deep Learning Radiomics; CDLR, Clinical Deep Learning Radiomics.

By integrating the clinical feature with the DLR feature through SVM algorithm, the CDLR model demonstrated enhanced and stable diagnostic performance (**Figures 4A, B**; **Table 2**).

**Table 2:** Training set and validation set performance metrics for predictive models.

Model	Cohort	AUC (95%CI)	Accuracy (95%CI)	Sensitivity (95%CI)	Specificity (95%CI)	NPV (95%CI)	PPV (95%CI)	p-value
Clinic	Training	0.846 (0.8116- 0.8806)	0.692 (0.6496- 0.7314)	0.5714 (0.5174- 0.6238)	0.943 (0.8953- 0.9697)	0.5138 (0.4565- 0.5708)	0.9543 (0.9155- 0.9758)	<0.001*
	Validation	0.828 (0.7478- 0.9073)	0.6393 (0.5541- 0.7246)	0.5376 (0.4363- 0.6390)	0.9655 (0.8991- 1.0000)	0.3944 (0.2807- 0.5080)	0.9804 (0.9423- 1.0000)	0.012*
Rad	Training	0.857 (0.8170- 0.8960)	0.7967 (0.7587- 0.8301)	0.772 (0.7237- 0.8141)	0.8481 (0.7839- 0.8957)	0.6411 (0.5741- 0.7031)	0.9137 (0.8748- 0.9413)	<0.001*
	Validation	0.845 (0.7702- 0.9205)	0.7213 (0.6359- 0.7932)	0.6559 (0.5549 -0.7445)	0.931 (0.7804- 0.9809)	0.4576 (0.337- 0.5834)	0.9683 (0.8914- 0.9913)	0.024
DTL	Training	0.911 (0.8824- 0.9402)	0.9261 (0.8994- 0.9461)	0.9392 (0.9080- 0.9603)	0.8987 (0.8418- 0.9367)	0.8765 (0.8170- 0.9186)	0.9508 (0.9215- 0.9695)	<0.001*
	Validation	0.867 (0.7970-0.9375)	0.8689 (0.7975-0.9176)	0.9140 (0.8393-0.9558)	0.7241 (0.5428-0.8530)	0.7241 (0.5428-0.8530)	0.9140 (0.8393-0.9558)	0.047
DLR	Training	0.986 (0.9756- 0.9972)	0.9548 (0.9326- 0.9700)	0.9544 (0.9261- 0.9722)	0.9557 (0.9114- 0.9784)	0.9096 (0.8563- 0.9445)	0.9782 (0.9557- 0.9894)	0.92
	Validation	0.897 (0.8410- 0.9521)	0.8197 (0.7420- 0.8778)	0.7957 (0.7028- 0.8651)	0.8966 (0.7361- 0.9642)	0.5778 (0.4330- 0.7103)	0.961 (0.8916- 0.9867)	0.072
CDLR	Training	0.987 (0.9771- 0.9963)	0.9630 (0.9423- 0.9765)	0.9635 (0.9373- 0.9790)	0.9620 (0.9196- 0.9825)	0.9268 (0.8765- 0.9576)	0.9814 (0.9601- 0.9915)	
	Validation	0.924 (0.8717- 0.9756)	0.8852 (0.8287- 0.9418)	0.8817 (0.8161- 0.9474)	0.8966 (0.7857- 1.0000)	0.7027 (0.5554- 0.8500)	0.9647 (0.9255- 1.0000)	

CI, confidence interval; Rad, radiomics; DTL, Deep Transfer Learning; DLR, Deep Learning Radiomics; CDLR, Clinical Deep Learning Radiomics; PPV, positive predictive value; NPV, negative predictive value. *p value of AUCs different between the model and CDLR in the training and validation cohort.

**Figure 5:**
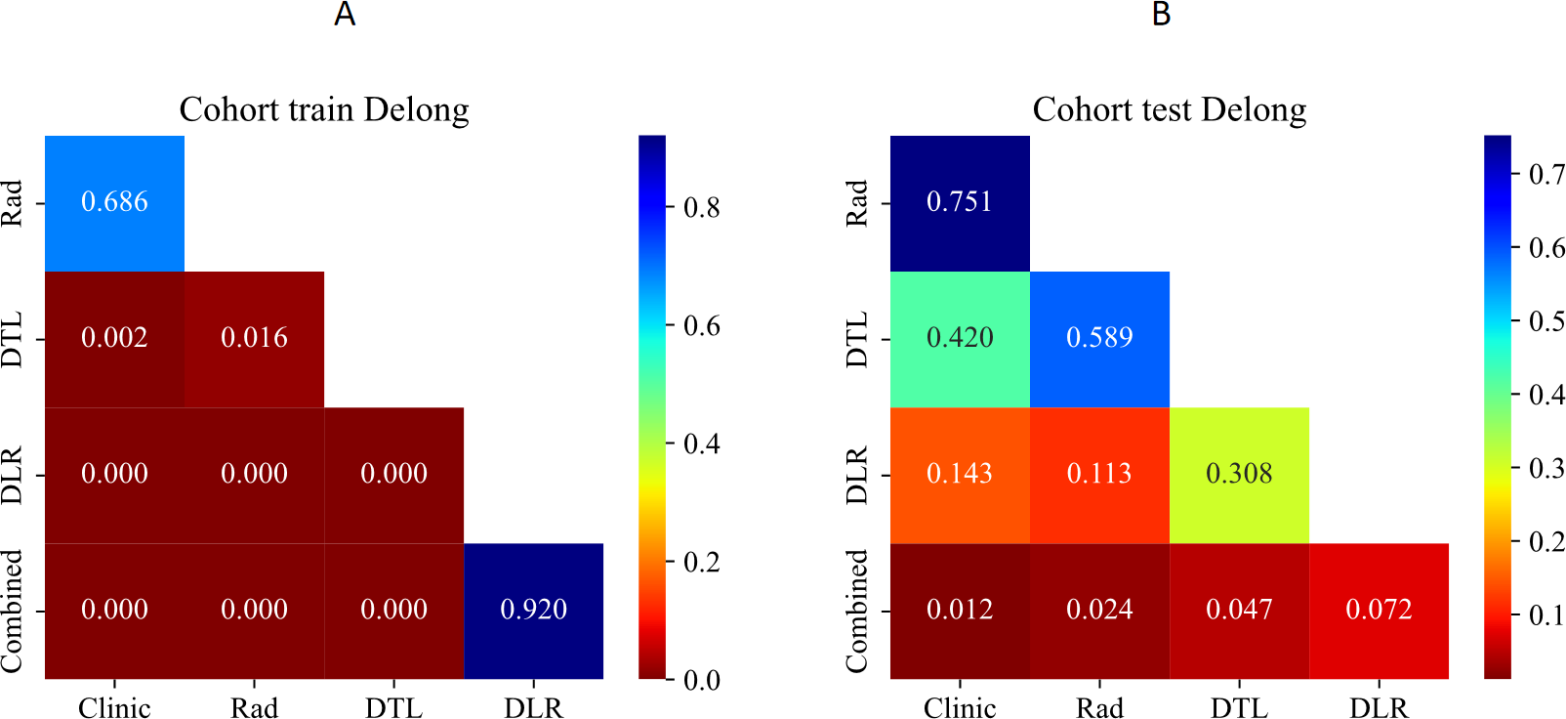
Delong’s test for comparison of CDLR model with different models. **(A)** The training set model, **(B)** The validation set model. Rad, radiomics; DTL, Deep Transfer Learning; DLR, Deep Learning Radiomics; Combined is CDLR Clinical Deep Learning Radiomics.

The DeLong test results showed that there were statistically significant differences between the combined model in the training set and the test set and the single clinical model, the Rad model, and the DTL model (p < 0.05),while there was no statistically significant difference when compared with the DLR model, as shown in **Figure 5**.

**Figure 6:**
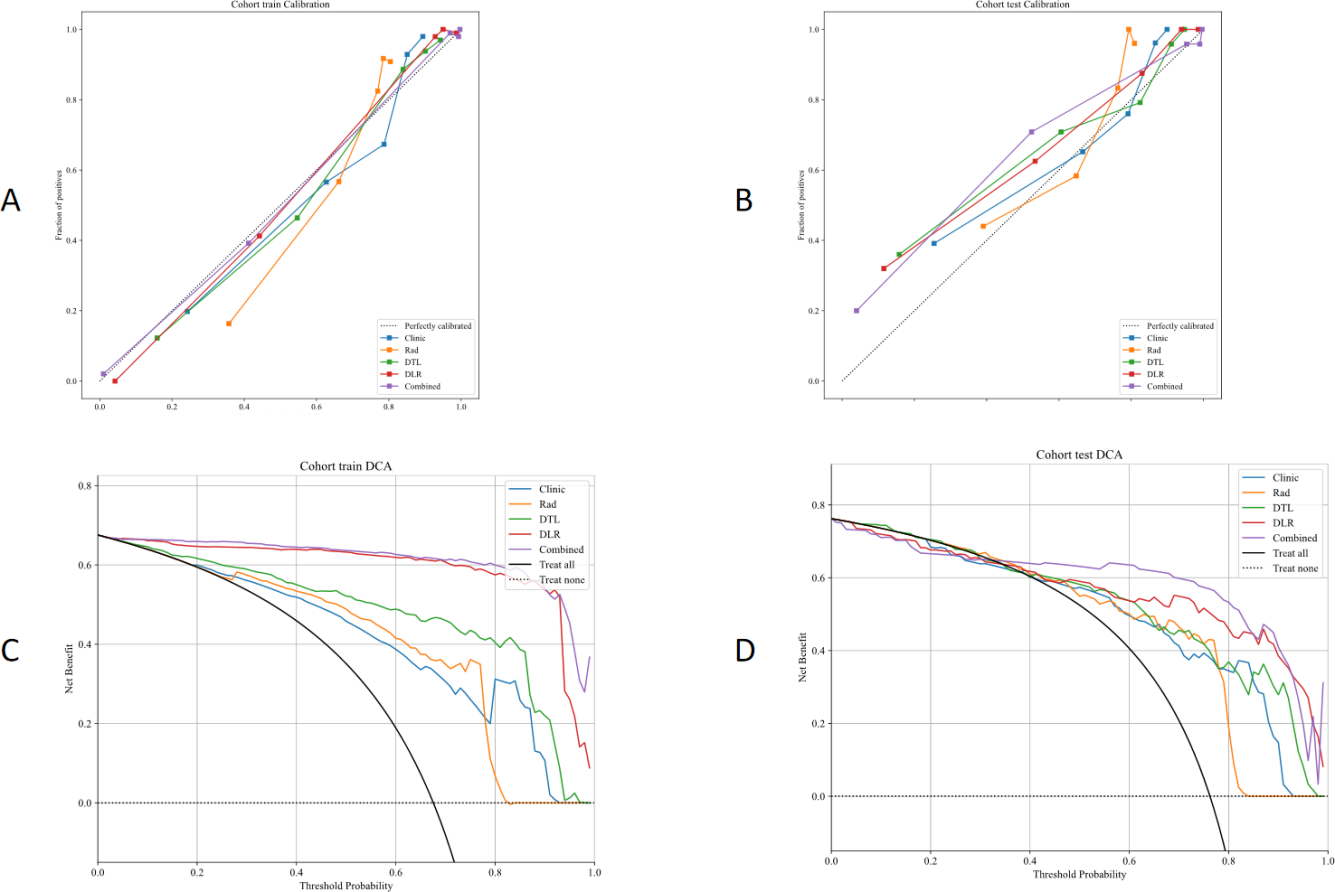
**(A, B)** The CC curves compare the performance of the clinical, Rad, DTL, DLR and CDLR models in the training set and validation set. **(C, D)** The DCA curve comparison between the clinical, Rad, DTL, DLR and CDLR models. CC, Calibration Curves; DCA, decision curve analysis; Rad, radiomics; DTL, Deep Transfer Learning; DLR, Deep Learning Radiomics; CDLR, Clinical Deep Learning Radiomics.

**Figure 7:**
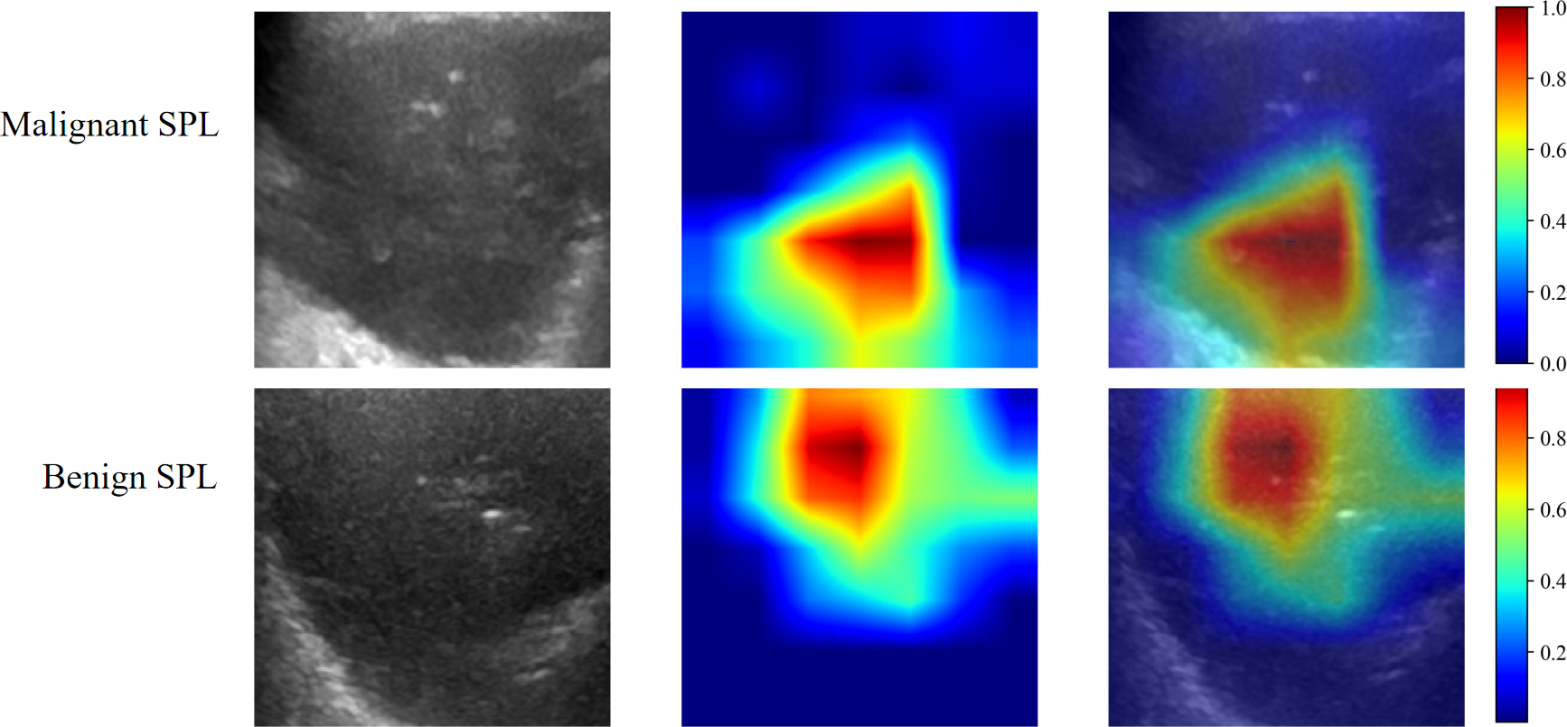
The convolutional neural network (CNN) model with Grad-CAM was used on the SPLs, the red area displayed the basis of decision-making of CNN.

CC curve (**Figures 6A, B**) showed that the CDLR model had the best overall calibration performance. It was almost consistent with the ideal state in the low-probability range. Although there was a slight underestimation in the medium and high probability ranges, the deviation is the smallest. The calibration deviations of pure clinical or single-feature models were relatively large, especially in terms of the prediction accuracy for high-risk populations. Decision curve analysis (DCA) results are shown in **Figures 6C, D**, which showed that the model has a clear clinical net benefit within the probability range of 0.05 to 0.4. The 0.1 to 0.3 range is the core effective interval, and the CDLR model performed the best within this range. This result provides a direct basis for the selection of the clinical decision threshold, supporting the recommendation of approximately 0.1 to 0.3 as the threshold range. **Figure 7** displayed the activation maps of the convolutional neural network used to distinguish between benign and malignant SPLs, highlighting key prediction regions in red.

**Figure 8:**
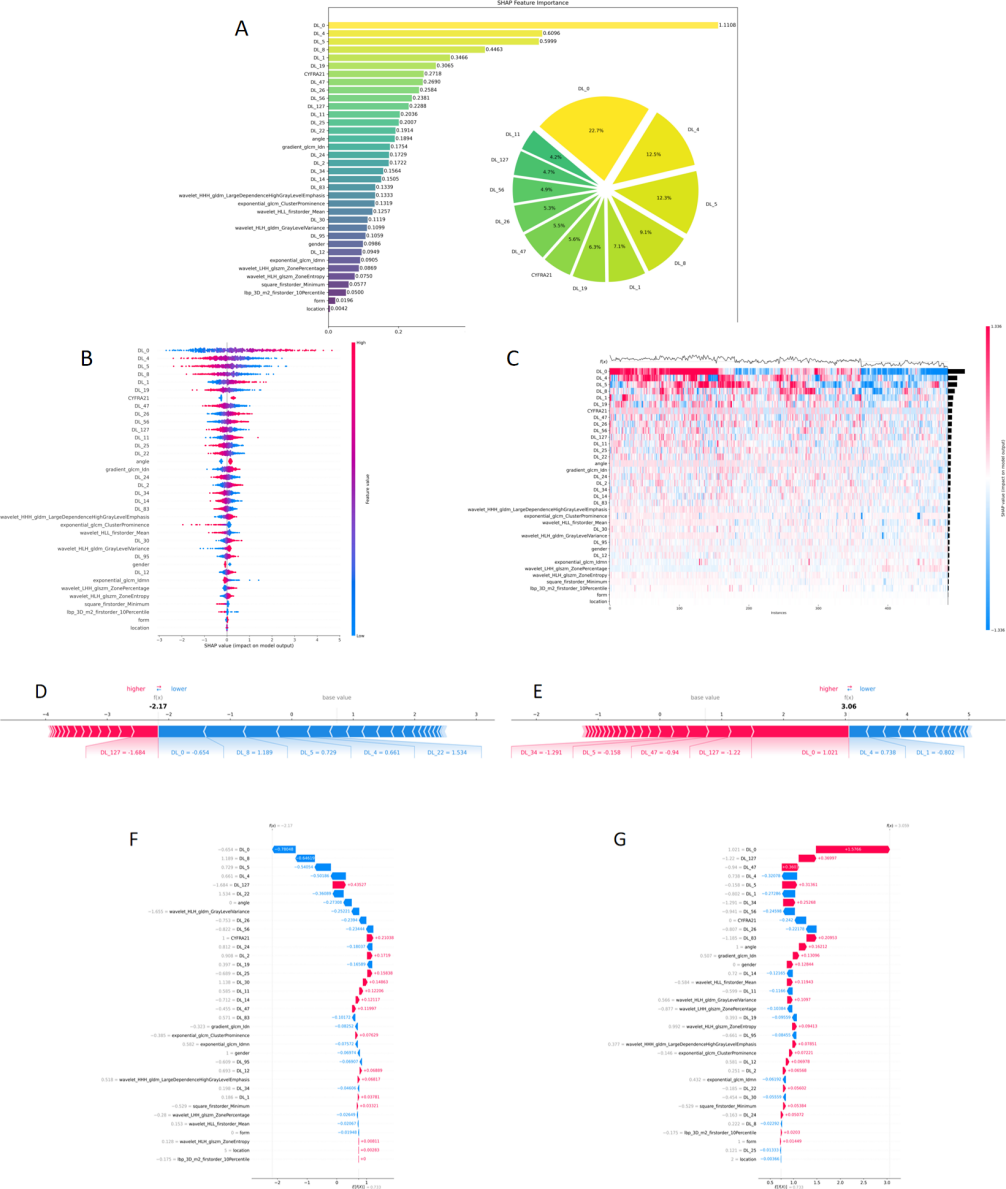
Explanatory analysis of CDLR Model using SHAP: **(A)** The SHAP bar chart (left) and pie chart (right); **(B)** The SHAP summary plot; **(C)** The SHAP heatmap; **(D, E)** The force plot interprets a single sample prediction; **(F, G)** The waterfall plot. CDLR, Clinical Deep Learning Radiomics.

The SHAP value was employed to interpret the CDLR model by calculating the contribution of each feature to the prediction of benign and malignant SPLs, providing intuitive and clear interpretative visualizations (**Figure 8**). The SHAP bar chart (**Figure 8A**) provides a global interpretation, displaying the importance ranking and proportional contribution of each feature, with the most important features positioned at the top. The results showed that DL features—DL_0, DL_4, and DL_5—were the three most contributing features. The SHAP summary plot (**Figure 8B**) illustrates the positive or negative impact of each feature on the prediction probability. Positive values indicate an increased likelihood of the predicted outcome, while negative values indicate a decreased likelihood. The horizontal axis represents SHAP values, each row corresponds to a feature, and each point represents a sample. Red indicates high feature values, while blue indicates low feature values. A wider distribution of feature points suggests greater influence, as observed for DL_0 in this study. The SHAP heatmap (**Figure 8C**) depicts the direction and magnitude of each feature’s influence across all model cases. The SHAP force plots (**Figures 8D, E**) and waterfall plots (**Figures 8F, G**) provide local explanations for individual predictions. Red represents a positive contribution to the prediction and increased malignancy risk, while blue represents a negative contribution and reduced malignancy risk. The SHAP base value represents the average of all the SHapley values of the predictive features, serving as the reference value in the SHAP additive explanation framework, while f(x) represents the final prediction probability. The case presented in **Figures 8D, F** has a model output value f(x) of -2.17, which is lower than the base value of 0.733. The contributing features are predominantly blue, pushing the prediction downward, and the final pathological result confirmed this case as benign. In contrast, the case shown in **Figures 8E, G** has an output value f(x) of 3.06, which is higher than the base value. The features exhibit prominent red positive contributions, increasing the likelihood of malignancy, and this case was correctly predicted as malignant.

## Discussion

This study aimed to develop and validate an ultrasound-based non-invasive machine learning model for predicting the benign or malignant nature of 609 patients with subpleural lung lesions. The CDLR model combined clinical, Rad, and DTL features and outperforms compared with one or two fusion models, offering superior stability. However, DTL and DLR models demonstrated superior value compared to the Rad model. The deep learning features highlighting their value in identifying key quantitative data that reflects the nature of the masses (15–17). To our knowledge, this is the first study to develop CDLR nomograms for predicting subpleural lung disease, specifically for identifying benign lesions and malignancies.

The study effectively mitigated the risk of overfitting through rigorous feature selection procedures which process ultimately resulted in a model with high repeatability and stability. For example, the performance discrepancy between the Rad model in the training set and validation set was minimal (AUC = 0.857 vs. 0.845), indicating the robustness of the selected radiomics features across cohorts. To optimize performance, expanding the sample size and refining training models will be essential. The combined CDLR model exhibited stability across all indicators, maintaining high performance in both the training and validation sets. Notably, its sensitivity (SEN = 0.871), specificity (SPE = 0.897), and accuracy (ACC = 0.877) in the validation set surpassed those of other models, indicating its strong ability to identify malignant lung tumors while minimizing misdiagnosis of benign cases. This demonstrates its potential clinical value for decision-making. The Delong test revealed significant differences in predictive efficacy between the combined model and other single models, highlighting the benefit of integrating multiple feature sets or models, which improves prediction accuracy (13, 18, 19). Additionally, calibration curve analysis and DCA showed that the combined model outperforms the others. Its calibrated probability and decision curve indicate its superior ability to accurately differentiate between benign and malignant SPLs, ensuring timely intervention for malignant lesions, enhancing treatment efficiency, and reducing unnecessary resource use for benign cases.

The lack of interpretability in ML models has led to a “black box” effect, which limits their clinical applicability (20, 21). To address this issue, SHAP was utilized to visualize the model’s prediction process, employing Shapley values from game theory to explain the contribution of each feature. By establishing the relationship between imaging features and biomarkers and conducting causal inference, the clinical value of this model in the biomedical field was further enhanced (14, 22, 23). The results indicate that, compared to Rad and clinical features, DL features play a decisive role in model predictions. Specifically, the average SHAP values of the four most significant DL features—DL_0, DL_4, DL_5, and DL_8—were 1.118, 0.6596, 0.5999, and 0.4681, respectively. The cumulative contribution of these features accounted for more than 50% of the total model prediction (22.7% + 12.5% + 12.3% + 9.1%). This outcome highlights the advantage of DL models in extracting high-order abstract features through multiple layers of nonlinear mapping. These models excel at capturing complex patterns, such as the texture changes in lesions and the spatial distribution of tumor micro-environments, which are directly related to the core biological essence of the tumor, aiding in the prediction of benign or malignant characteristics (24, 25). Traditional Rad features, such as gradient_glcm_ldn and wavelet_HHH_glcm_LargeDependenceHighGrayLevelEmphasis, quantify tumor edge irregularity and texture changes after wavelet transformation, providing essential details for differentiating benign from malignant tumors. These findings are consistent with prior conclusions regarding the effectiveness of traditional radiomics in the quantitative analysis of medical images, where manually designed features capture physiological and pathological correlations within the images (26–28). Although the overall contribution of clinical features is relatively low, significant variability exists within them. Basic clinical features, such as lesion location, shape, and gender, generally show SHAP values below 0.1. However, biological markers like CYFRA21–1 demonstrated a SHAP value of 0.2718, significantly higher than other clinical features. This suggests that CYFRA21-1, despite being a clinical indicator with clear biological relevance, still retains an independent contribution within the ML model.

By quantifying the contribution of features to the prediction, the study shifted from “black box prediction” to “interpretable decision-making.” Furthermore,. clinicians can understand the contribution of specific lesion features to being classified as benign or malignant through SHAP values. The SHAP base value represents the average of all the SHapley values of the predictive features, serving as the reference value in the SHAP explanation framework. In our study, the SHAP baseline value of 0.733 represents the average predicted risk probability of the model for all lesions. For a single lesion, when the cumulative SHAP value of its feature combinations is lower than this baseline value, it tends to exhibit a benign feature pattern; when the cumulative value is higher than this baseline value, it tends to exhibit a malignant feature pattern. It should be noted that this baseline value is used to explain the direction of feature contribution, rather than as a clinical decision threshold (29).

Subpleural lung lesions often appear spherical or nearly spherical on ultrasound, forming an obtuse angle with the chest wall, which serves as an important independent risk factor for malignant tumors (30, 31). Tumor markers like CYFRA21–1 play a key role in identifying lung tumors (32). Our findings align with these previous studies: the lesion was located in the left upper lobe of the lung, was spherical in shape, formed an obtuse angle with the chest wall, lacked bronchial signs, and exhibited an abnormal elevation of CYFRA21-1, which served as an independent predictor for subpleural lung tumors. Notably, men showed a protective OR of 0.338 (*p* < 0.001), indicating a lower risk of lung cancer compared to women. This observation is inconsistent with the 2022 Global Cancer Statistics Report (1). Potential reasons for this discrepancy include the higher number of male patients in our study compared to female patients and the lack of analysis on female-specific factors such as smoking and hormone replacement therapy, which may have overestimated the gender difference. Previous research has demonstrated that, under equivalent smoking exposure, the risk of lung cancer in female smokers is more than twice that in male smokers, and hormone replacement therapy has been associated with lung cancer incidence, median survival, and mortality rates (33, 34).

To improve the interpretability of the model, the Grad-CAM visualization technique was employed to generate heat-maps, highlighting the image regions that are crucial in the classification decision. This approach effectively guides clinical doctors in identifying the relevant areas of focus (13, 35). By combining SHAP, which quantifies feature contributions, with Grad-CAM, which identifies key image regions, this study explained the model’s decision-making from both “what is important” and “where” perspectives. This dual approach offers doctors intuitive evidence to understand the model’s decision logic, thereby enhancing trust in the prediction results of the DTL model. Furthermore, when integrated into ultrasound devices, this could facilitate real-time lesion marking and risk prediction, offering a novel diagnostic method for disease detection.

However, this study has several limitations. As a retrospective analysis, selection bias is inevitable, While only conventional gray-scale ultrasound was employed, and no techniques such as ultrasound contrast imaging or elasticity measurement were used. Lastly, a prospective multi-center study will be conducted and more modalities of ultrasound techniques to further validate the proposed model.

## Conclusion

The clinical deep learning radiomics (CDLR) ultrasound imaging biomarker atlas presented in this study demonstrates promising results in predicting the benign and malignant nature of subpleural lung lesions. This is of critical importance for early diagnosis and treatment planning. The technology can effectively reduce unnecessary biopsies or surgeries for non-essential masses, minimizing excessive medical intervention and providing a solid foundation for achieving precise tumor treatment.

## Data Availability

The raw data supporting the conclusions of this article will be made available by the authors, without undue reservation.
