# Supplementary material for: Ultrasound-based deep learning radiomics for the differential diagnosis of benign and malignant subpleural pulmonary lesions
Source: Front Oncol. 2026 Mar 16;16:1786674. doi: 10.3389/fonc.2026.1786674 (PMC13033524; doi:10.3389/fonc.2026.1786674)
Supplement: Supplementary file 1 [file DataSheet1.docx]

**Supplementary Material**

Supplementary Material Table E1

Table E1 Disease Pathological Names and Case Numbers Included in SPL in This Study

| Variable classification | Pathological name | numbers |
| --- | --- | --- |
| Malignant (422) | Adenocarcinoma | 266 |
|  | Squamous carcinoma | 93 |
|  | Small cell carcinoma | 22 |
|  | Sarcomatoid carcinoma | 6 |
|  | Lymphoma | 11 |
|  | Mixed carcinoma | 10 |
|  | Other sources of malignant tumors | 14 |
| Benign（187） | Pneumonia | 120 |
|  | Pulmonary fungal infection | 24 |
|  | Tuberculosis of the lungs | 23 |
|  | Lung abscess | 5 |
|  | Inflammatory pseudotumor | 8 |
|  | Other benign lesions | 7 |

Supplementary Material E2

Examination method: Patients were positioned in the supine position, prone position, left/right lateral position according to the location of the lesion. The examiner gently moved and rotated the probe, and when necessary, instructed the patients to hold their breath. Careful scanning and recording of the location, maximum left-right diameter, maximum anterior-posterior diameter, shape, angle with the chest wall, internal bronchial signs, and internal blood flow were conducted. The location of the lesion was specifically divided into left upper lobe of the lung, left lower lobe of the lung, right upper lobe of the lung, right middle lobe of the lung, and right lower lobe of the lung based on the anatomical location. The shape of the lesion was classified based on ultrasound images as spherical and wedge-shaped; the angle between the lesion and the chest wall was classified as acute angle and obtuse angle. The classification criteria were: when the angle between both sides was acute angle, it was classified as acute angle; when one or both sides were obtuse angle, it was classified as obtuse angle. The internal bronchial signs and internal blood flow in this study were both binary classified: presence/absence of bronchial signs, presence/absence of blood flow signals.

Supplementary Material E3

The specific PyRadiomics configuration settings are as follows:

Version: Python (version 3.7.12) ;

Image discretization: Set the bin width to 5 HU and increase the voxel value by 1000 to avoid negative values in the calculation of first-order features.

Normalization: Enabled, with a ratio of 1000 to ensure consistency of the scan results.

Resampling: Use sitkNearestNeighbor interpolation, with a isotropic spacing of 3×3×3 mm, and a padding distance of 10 voxels for the multi-scale LoG filter.

Image types: Original image, LoG ( sigma: [1.0, 2.0, 3.0]), wavelet transform, LBP3D, exponential function, square function, square root function, logarithmic function, and gradient transformation.

Feature categories: Shape, first-order features, GLCM (excluding 22 features of the sum and average values), GLRLM, GLSZM, GLDM, and NGTDM.

Reproducibility enhancement:

By providing these detailed information, we aim to enable other researchers to precisely replicate our feature extraction process. Additionally, we provide a complete configuration file in YAML format as a supplementary file "exampleUS.yaml" to ensure complete transparency of the information.

Radiomics features included: (1) fourteen 2D shape-based features, (2) 306 first-order features, and (3) texture features, such as those derived from the gray-level co-occurrence matrix (GLCM, n = 374), gray-level dependence matrix (GLDM, n = 238), gray-level run length matrix (GLRLM, n = 272), gray-level size zone matrix (GLSZM, n = 272), and neighboring gray tone difference matrix (NGTDM, n = 85). This resulted in a total of 1,561 radiomics features.

For DL feature extraction, there were the following four main steps: (1) Data preprocessing: Preprocess the labeled lesion region ROI, including cropping it to 224×224 pixels and normalizing the pixel gray values, etc.; (2) Model loading and adjustment: Extract the output of the average pooling layer of ResNet-50 as the feature. In order to enable ResNet-50 to extract deep feature information of the lesion from a small amount of ultrasound data, a transfer learning method is selected. All experiments were conducted with a fixed random seed (random_state=0), with training parameters including batch size of 32, 50 epochs, initial learning rate of 0.01, and SGD optimizer. Features were extracted from the global average pooling layer (avgpool), and all procedures were implemented using the Onekey platform to ensure reproducibility; (3) Feature extraction: Input the preprocessed image into the adjusted ResNet-50 to generate 2048 features for each image; (4) Feature compression and dimensionality reduction: This study adopts the principal component analysis (PCA) dimensionality reduction technology to perform linear dimensionality reduction compression on the original 2048-dimensional features. During the compression process, strictly follow the constraint condition that the number of target features must be less than the number of samples to avoid contradictions in the covariance matrix, and finally retain the feature vectors corresponding to the 128 largest eigenvalues to improve the generalization ability of the model and reduce the risk of overfitting.

Supplementary Material Table E4

Table E4 Distribution of Clinical Parameters in the Training and Validation Sets

| Variate | Training （n=487） | |  | Validation（n=122） | | *p* |
| --- | --- | --- | --- | --- | --- | --- |
|  | Benign | Malignant | *p* | Benign | Malignant |  |
| Age | 53.40±14.20 | 62.81±11.49 | <0.001 | 55.83±13.52 | 61.39±13.22 | 0.052 |
| Gender |  |  | 0.245 |  |  | 0.465 |
| Female | 68 | 122 |  | 9 | 38 |  |
| Male | 90 | 207 |  | 20 | 55 |  |
| Smoking history |  |  | 0.019 |  |  | 0.746 |
| No smoking | 94 | 157 |  | 16 | 46 |  |
| Smoking | 64 | 172 |  | 13 | 47 |  |
| Anatomical location of the lesion |  |  | 0.182 |  |  | 0.575 |
| Left upper | 28 | 87 |  | 4 | 23 |  |
| Left lower | 38 | 74 |  | 8 | 21 |  |
| Right upper | 35 | 77 |  | 5 | 22 |  |
| Right middle | 13 | 23 |  | 3 | 7 |  |
| Right lower | 44 | 68 |  | 9 | 20 |  |
| Left-right diameter (cm) * | 4.85±2.37 | 6.57±3.17 | <0.001 | 3.76±1.83 | 6.27±2.74 | <0.001 |
| Anterior-posterior diameter (cm) * | 3.54±1.68 | 5.09±2.51 | <0.001 | 3.00±1.57 | 5.44±2.54 | <0.001 |
| Shape * |  |  | <0.001 |  |  | 0.003 |
| Spherical | 107 | 102 |  | 19 | 30 |  |
| Wedge-shaped | 51 | 227 |  | 10 | 63 |  |
| Angle * |  |  | <0.001 |  |  | <0.001 |
| Acute | 92 | 63 |  | 15 | 16 |  |
| Obtuse | 66 | 266 |  | 14 | 77 |  |
| Bronchial sign within the lesion |  |  | <0.001 |  |  | 0.08 |
| No | 81 | 227 |  | 17 | 72 |  |
| Yes | 77 | 102 |  | 12 | 21 |  |
| Blood flow within the lesion* |  |  | 0.012 |  |  | 0.015 |
| No | 70 | 106 |  | 18 | 32 |  |
| Yes | 88 | 223 |  | 11 | 61 |  |
| CA125 |  |  | <0.001 |  |  | 0.443 |
| Normal | 113 | 158 |  | 21 | 58 |  |
| Abnormal | 45 | 171 |  | 8 | 35 |  |
| CYFRA21-1* |  |  | <0.001 |  |  | <0.001 |
| Normal | 127 | 96 |  | 24 | 27 |  |
| Abnormal | 31 | 233 |  | 5 | 66 |  |

注：* For log
